# Evaluating Wagner Oxidation Criteria for Protective Al_2_O_3_ Scale Formation in Ni-Based Superalloys

**DOI:** 10.1007/s11085-023-10163-5

**Published:** 2023-06-26

**Authors:** J. W. X. Wo, M. C. Hardy, H. J. Stone

**Affiliations:** 1 Department of Materials Science and Metallurgy, University of Cambridge, 27 Charles Babbage Road, Cambridge, CB3 0FS UK; 2 Rolls-Royce Plc, PO Box 31, Derby, DE24 8BJ UK

**Keywords:** Ni-based superalloys, Oxidation, Modelling, Electron microscopy

## Abstract

An assessment is made of the Wagner transition criteria for predicting the formation of a continuous Al_2_O_3_ scale in Ni-based superalloys. Predictions are compared with data from an experimental Ni-based superalloy as well as commercial superalloys for which published data are available. The methodology was generally successful in predicting the transition temperature of the commercial superalloys but underpredicted the transition temperature of the experimental superalloy by approximately 50–100 °C. The difference in the transition temperature of the experimental superalloy to form a continuous Al_2_O_3_ scale is primarily attributed to a complex oxide subscale that increased the internal volume fraction of oxide and led to reduced oxygen ingress. The sensitivity and limitations of the methodology are discussed, and recommendations are made to refine the methodology to facilitate the interpretation of oxidation behaviour in polycrystalline Ni-based superalloys.

**Supplementary Information:**

The online version contains supplementary material available at 10.1007/s11085-023-10163-5.

## Introduction

Ni-based superalloys used for high-temperature applications must resist surface degradation through oxidation. This is typically achieved by selecting alloy compositions that automatically form a passive oxide layer on the surface that hinders further oxidation damage. Al and Cr are well known for producing protective oxides and are particularly suitable for selective oxidation [1–4]. Giggins and Pettit [1] identified three oxidation types in Ni–Cr–Al alloys: Type I alloys which form a non-protective external NiO scale with a discontinuous subscale of alumina (Al_2_O_3_) and chromia (Cr_2_O_3_) intrusions; Type II alloys which form a continuous external Cr_2_O_3_ scale with subscale discontinuous Al_2_O_3_ intrusions; and Type III alloys which form a continuous Al_2_O_3_ scale externally or underneath an external Cr_2_O_3_ scale.

Polycrystalline Ni-based superalloys used for turbine discs are traditionally designed to form Cr_2_O_3_ scales (Type II) for oxidation resistance because they experience relatively lower service temperatures than their single-crystal turbine blade counterparts. At lower temperatures, Cr_2_O_3_ forms a continuous scale faster than Al_2_O_3_ and provides superior corrosion resistance. However, Cr_2_O_3_ is unsuitable at higher temperatures as it can form volatile compounds (e.g. CrO_3_). This results in significant mass loss at temperatures beyond 1000 °C, and at lower temperatures when the gas velocity is sufficiently high. Critically, this instability of Cr_2_O_3_ at higher temperatures, combined with relatively slower elemental diffusion in Al_2_O_3_ [5], suggests that polycrystalline superalloys need to be designed as Al_2_O_3_ formers if the maximum operating temperatures are to be increased to 800 °C and beyond. However, experimental determination of multi-component superalloy compositions that form continuous Al_2_O_3_ scales can be a laborious trial-and-error process. Due to the vast number of possible compositions, experimental assessment is impractical [6]. It is therefore attractive to employ computer-aided models to determine superalloy compositions with good oxidation resistance by predicting the proclivity of a given superalloy composition to preferentially form a protective oxide (e.g. Cr_2_O_3_ or Al_2_O_3_). To achieve this, various modelling approaches have been proposed to describe the internal oxidation processes in relatively simple alloy systems [7–9]. However, a complete understanding of the underlying oxidation mechanisms of a superalloy requires a thorough analysis of the interplay between numerous factors (e.g. temperature, diffusion chemistry, oxidising gases, time, alloy compositions, etc.). To this end, several attempts have been made to develop modelling approaches that consider these factors in greater detail [10–12].

Several modelling strategies have been based on Wagner’s theory [13], which extended Darken’s analysis [14] for the transition from internal to external oxidation by assuming that the transition will occur if the solute concentration is sufficiently high such that the volume fraction of the internal oxides surpasses a critical value. Assuming a binary A–B system, Wagner predicted that the transition would occur when the concentration of the oxide-forming element is given by:

1 $$N_{{{\text{Al}}}}^{\left( 1 \right)} \ge \left[ {f\left( {\frac{{V_{{\text{m}}} }}{{V_{{{\text{ox}}}} }}} \right)\pi \frac{{N_{{\text{O}}}^{{\text{S}}} D_{{\text{O}}} }}{{2\nu D_{{{\text{Al}}}} }}} \right]^{\frac{1}{2}}$$

where $N_{{{\text{Al}}}}^{\left( 1 \right)}$ is the minimum critical concentration of solute B (assumed to be Al in this study) required for external oxidation (mol fraction), $f$ is the volume fraction of internal oxide, $V_{{\text{m}}}$ and $V_{{{\text{ox}}}}$ are the respective molar volumes of the alloy and oxide (cm^3^/mol), $N_{{\text{O}}}^{{\text{S}}}$ is the solubility of oxygen in the alloy (mol fraction), $D_{{\text{O}}}$ is the diffusivity of oxygen in the alloy (cm^2^/s), $D_{{{\text{Al}}}}$ is the diffusivity of the solute B (Al) in the alloy (cm^2^/s), and $\nu$ is the stoichiometric ratio of oxygen to solute atoms B (i.e. 1.5 for Al_2_O_3_). The selection and calculation of the diffusivities for both the oxidant and solute elements as well as the oxidant solubility are crucial [10].

Rapp [15] experimentally verified Wagner’s theory in a Ag–In system at 550 °C and found the critical volume fraction of In_2_O_3_ to be 0.3 and the minimum critical concentration of In to be 0.15 (mol fraction). While the experimental results were in reasonable agreement with Wagner’s theory, the critical volume fraction was proposed to be highly dependent on composition, highlighting the simplifying assumptions of Wagner’s theory. Wagner’s theory was experimentally assessed in Ni–Al alloys [16] and significantly underpredicted experimental Al concentrations required for protective oxide formation. However, it was also found that the theory was in reasonable agreement with experimental Al concentrations in ternary Ni–Cr–Al alloys at 1200 °C.

If the solute concentration of a given alloy satisfies Eq. (1), it suggests that achieving a continuous and protective oxide scale is theoretically possible. However, the ability of the alloy to sustain the steady growth of the established continuous oxide scale is not necessarily automatic. By assuming that the concentration and diffusion of solute B must be equal to or higher than its consumption rate to form the oxide, Wagner [17] derived the following additional criterion:

2 $$N_{{{\text{Al}}}}^{\left( 2 \right)} \ge \frac{{V_{{\text{m}}} }}{{2\nu M_{{\text{O}}} }}\left[ {\frac{{\pi k_{{\text{p}}} }}{{D_{{{\text{Al}}}} }}} \right]^{\frac{1}{2}}$$

where $N_{{{\text{Al}}}}^{\left( 2 \right)}$ is the minimum critical concentration of solute B (e.g. Al) required for maintaining external scale formation, $k_{{\text{p}}}$ is the parabolic rate constant for the growth of the continuous oxide scale (g^2^/m^4^s), and $M_{{\text{O}}}$ is the molar mass of the oxidant (i.e. 16 g/mol for monoatomic oxygen). Similar to Eq. (1), the diffusivity of solute B (Al), $D_{{{\text{Al}}}}$, is assumed to be independent of concentration. The criterion assumes that oxide scale growth follows parabolic kinetics. Furthermore, solvent atoms are assumed to be insoluble in the oxide scale and no recession of the alloy/oxide interface occurs. Previous studies have used Eq. (2) to understand the oxidation behaviour of Fe–Cr alloys in water vapour environments [18, 19], which found that $N_{{{\text{Al}}}}^{\left( 2 \right)}$ values were generally lower than $N_{{{\text{Al}}}}^{\left( 1 \right)}$ and, unlike Eq. (1), increased with temperature for some alloys. It is also noted that both studies used $f$ = 0.3, as reported in [15].

The potential effect of an existing outer oxide scale on the minimum critical solute concentration has also been studied [20]. It was found that the transition was more difficult to achieve due to the reduced enrichment of the less noble component in the internally oxidised region in the presence of an outer scale, which requires a higher minimum critical solute concentration to overcome the growth rates of the outer scale. To address the alloy-specific dependency of the critical volume fraction of oxide, a quantitative approach was developed [21] in a Ni–Cr–Al alloy by considering an “effective” diffusion coefficient of oxygen in the alloy and the constantly increasing barrier effect of internal oxide precipitates. However, it was also shown experimentally that the calculated critical volume fraction was generally an overestimate [21].

An expanded version of Smith’s model [22] was developed and applied by Guan et al. [23] to establish the solubility of oxygen in ternary alloys as a criterion for predicting the minimum Al concentrations required to form Al_2_O_3_ in Ni–Cr–Al alloys at 1100 °C and 1200 °C. The authors found that the required Al concentrations ranged from 2 to 6 at% depending on the temperature, which agreed with Nesbitt in [16]. Both studies observed that the addition of Cr has a significant beneficial effect on forming Al_2_O_3_ through the reduction of overall oxygen solubility in the alloy.

It is also well known that Al and Cr additions offer synergistic benefits to oxidation performance. Specifically, the presence of Cr can promote the oxidation of Al (i.e. formation of Al_2_O_3_) at lower Al concentrations, inhibiting the diffusion of oxygen through the alloy by acting as an oxygen “getter” [1]. The reverse has also been observed where Al similarly promotes the formation of Cr_2_O_3_ [24]. However, the situation is complicated by other alloying elements having potential effects on selective oxidation. For example, Mo and W can act beneficially as oxygen getters and detrimentally by inhibiting diffusion of Al to the superalloy surface [25]. These reasons suggest that Al_2_O_3_-forming polycrystalline superalloys, supported by careful additions of Cr, are the preferred option for higher temperature oxidation resistance.

There is a consensus that the reliable prediction of the criteria required to form a continuous Al_2_O_3_ scale in multi-component superalloys is challenging, and various attempts have shown mixed results. As such, the purpose of this paper is to evaluate the classic Wagner criteria for predicting the critical solute concentration required to transition from internally oxidised inclusions to an external scale as well as to sustain protective scale growth for Ni-based superalloys. The criteria are tested against an experimental superalloy, and its applicability to several commercial superalloys is discussed. The resulting observations are used to quantify the differences between the Wagner criteria predictions and experimental results so that aspects of the model can be identified for further refinement and potential use in interpreting oxidation behaviour in Ni-based superalloys.

## Methods

### Materials

The composition of the experimental Ni-based superalloy (Alloy X) is outlined in Table 1. The concentrations of Al (11.30 at%) and Cr (12.74 at%) were chosen to be the median of the range of observed Al and Cr concentrations in commercial superalloys. *Thermo-Calc 2021b (Thermo-Calc),* a thermodynamic modelling software package, was used to determine the concentrations of other elements for minimising topologically-close-packed (TCP) phase formation and maximising alloy strength. Mo was added for solid solution strengthening, Nb/Ta were added to promote the formation and strengthening of the γ′ phase, Co was included for solid solution strengthening and minimisation of stacking fault energy, and W was added to improve solution strengthening/creep resistance. In this alloy, Ti was limited to avoid negative oxidation effects while still being sufficient to promote γ′ formation and increase APB energy [26]. A comparatively high concentration of Mn was included for enhanced oxidation resistance, while concentrations of B, C, and Zr were maintained at similar levels compared with the other commercial alloys.

**Table 1:** Nominal alloy compositions (at%) considered in this study. Compositions obtained from [1, 26–29]

at%	Ni	Al	B	C	Co	Cr	Hf	Mn	Mo	Nb	Re	Ta	Ti	W	Zr
GP type I	92.32	2.14	–	–	–	5.54	–	–	–	–	–	–	–	–	–
Waspaloy	56.52	2.94	0.03	0.28	12.99	21.27	–	–	2.36	–	–	–	3.56	–	0.03
GP Type II	64.02	4.10	–	–	–	31.88	–	–	–	–	–	–	–	–	–
RR1000	50.89	6.35	0.11	0.14	17.93	16.48	0.16	–	2.98	–	–	0.63	4.30	–	0.04
IN 738	59.48	7.00	–	0.76	8.11	17.55	–	–	1.03	0.53	–	0.54	4.22	0.81	–
Alloy X	49.85	11.30	0.20	0.15	19.36	12.74	–	0.59	1.79	1.02	–	1.63	0.04	1.27	0.06
GP type IIIA	57.70	11.77	–	–	–	30.53	–	–	–	–	–	–	–	–	–
CMSX-4	63.75	12.59	–	–	9.26	7.58	0.03	–	0.38	–	0.98	2.18	1.27	1.98	–
PWA 1484	63.19	12.91	–	–	10.56	5.98	0.03	–	1.30	–	1.00	2.99	–	2.03	–
Rene N5	63.47	13.90	–	–	8.21	8.14	0.07	–	1.26	–	0.97	2.34	–	1.64	–
GP type IIIB	72.41	17.50	–	–	–	10.09	–	–	–	–	–	–	–	–	–

The superalloys for which published oxidation data at 800 °C were available and selected for analysis were separated into two categories: Rene N5, PWA 1484, and CMSX-4 (known Al_2_O_3_ formers); Waspaloy, IN 738, and RR1000 (known Cr_2_O_3_ formers). For further validation, the oxidation performance at 1000 °C of a quartet of ternary Ni–Cr–Al alloys [Type I: Ni–5Cr–1Al (wt%), Type II: Ni–30Cr–2Al (wt%), Type IIIA: Ni–30Cr–6Al (wt%), Type IIIB: Ni–10Cr–9Al (wt%)] from Giggins and Pettit (GP) [1] was also considered.

Samples of Alloy X were prepared by melting elements of 99.9% purity or higher with an Edmund Bühler Arc Melter. After melting, the samples were super-solvus solutioned at 1200 °C for 6 h, followed by air cooling. The samples were subsequently precipitate-aged at 843 °C and 800 °C for 2 h at each temperature. The actual composition of Alloy X was subsequently measured with large-area energy dispersive X-ray (EDX) analysis and averaged over three representative regions with approximate field-of-view dimensions of 52 × 40 μm^2^ and an acquisition time of 10 min per region. This information is accessed in Supplementary Information.

### Furnace Exposure Oxidation Experiments

Furnace exposure oxidation experiments were carried out on Alloy X samples to compare with the predictions made by the Wagner model. The samples were elliptical cross sections of the arc-melted bar with approximate 13 × 9 × 2 mm^3^ dimensions, polished to a 1-µm diamond finish. The samples were individually exposed to air from 750 to 1050 °C for 100 h in a Carbolite CWF1100 laboratory furnace.

### Thermogravimetric Analysis

A thermogravimetric analysis (TGA) measurement of Alloy X was performed using a Setaram Setsys Evolution 18 apparatus to study the specific mass change associated with oxidation. The TGA experiment was carried out at 800 °C for 100 h in air. The sample dimensions were approximately 13 × 9 × 1 mm^3^. Stereographs of the sample faces and thickness measurements were acquired and analysed with ImageJ processing software to obtain surface areas for calculating specific mass changes. All sample faces and sides were polished to a 1-µm diamond finish. Before the TGA experiment, the sample was cleaned in acetone followed by ethanol in an ultrasonic bath and dried for at least 24 h. The sample was first heated from 20 to 200 °C at 10 °C/min and held for 10 min to stabilise. The sample was then heated from 200 to 775 °C at 20 °C/min. To avoid overshooting the target temperature, the sample was heated from 775 to 800 °C at 5 °C/min. During the cooling process, the sample was cooled from 800 to 20 °C at 35 °C/min.

### Oxide Analyses

The oxidised samples were sectioned with a SiC saw using a low-impact dry-cut method to minimise damage to the oxide layers on the sample surfaces. The sectioned samples were mounted in phenolic resin and subsequently polished with standard SiC grinding papers culminating in a 0.06-µm colloidal silica suspension surface finish.

Analysis of the polished cross sections was carried out in a Zeiss GeminiSEM 300 scanning electron microscope (SEM) operated at 20 kV and a working distance of approximately 8.5 mm. The instrument was also equipped with an Oxford Instruments EDX detector for compositional analyses of microstructural features. Oxide cross sections were examined in backscattered electron (BSE) mode, and EDX elemental concentration maps were acquired in parallel.

### Equations and Assumptions

Equation (1) was used in all instances to calculate the minimum critical concentration of Al required to form a continuous Al_2_O_3_ scale at 800 °C in air. Several approaches were taken to calculate the parameters in Eq. (1) and are outlined here.

Equation (1) assumes a binary A–B system. While the investigated alloys are multi-component systems, it was idealised that the main participants in the oxidation processes consist of either Cr/Al within a predominantly Ni base. In addition, no external scale was assumed to be initially present following the assumptions made by Wagner in the derivation of Eq. (1) [13].

*Thermo-Calc* was used to calculate the molar volumes, $V_{{\text{m}}}$ (cm^3^/mol), of the investigated alloys at 800 °C and 1000 °C using the TCNi8 v8.2 database. The molar volume, $V_{{{\text{ox}}}}$, of Al_2_O_3_ was calculated from standard density and molecular weight values from [30] to be 25.575 cm^3^/mol.

By approximating the bulk concentration of the investigated alloys as mostly Ni, the solubility of O, $N_{{\text{O}}}^{{\text{S}}}$, and the diffusion coefficient of O in the alloy, $D_{{\text{O}}}$ (cm^2^/s), were calculated with formulations from Park and Altstetter [31] in solid Ni from 800 to 1000 °C:

3 $$N_{{\text{O}}}^{{\text{S}}} = 8.3e^{{\left[ {\frac{{ - 55\,\frac{{{\text{kJ}}}}{{{\text{mol}}}}}}{RT}} \right]}}$$

4 $$D_{{\text{O}}} = 1.7 \times 10^{ - 5} e^{{\left[ {\frac{{ - 90\,\frac{{{\text{kJ}}}}{{{\text{mol}}}}}}{RT}} \right]}}$$

where $R$ is the universal gas constant (J/mol K) and $T$ is the temperature (K). As a baseline, the value of $D_{{{\text{Al}}}}$ (cm^2^/s) of Al in solid nickel from 800 to 970 °C was also calculated with a formulation from Allison and Samelson [32]:

5 $$D_{{{\text{Al}}}} = 1.1e^{{\left[ {\frac{{ - 249\frac{{{\text{kJ}}}}{{{\text{mol}}}}}}{RT}} \right]}}$$

where the diffusivities $D_{{\text{O}}}$ and $D_{{{\text{Al}}}}$ were assumed to be independent of other elements present in the alloys (i.e. approximating the matrix as pure Ni). For comparison, the total diffusivity of Al in the alloys, $D_{{{\text{Al}}}}^{{\text{T}}}$, was also calculated in *Thermo-Calc* by utilising the TCNi8 v8.2 and MOBNi3 v3.2 databases. By setting the diffusing element to Al, the reference element to Ni (being the majority element in these alloys), and the gradient element to each component in the alloy, the diffusion coefficient of Al with reference to Ni in the presence of a gradient for each alloy component was computed. The total diffusivity of Al was taken as the sum of these individual diffusion coefficients.

The volume fraction of internal oxide, $f$, was selected to be 0.3, following the work of Rapp [15], and is considered a conventional value in the literature. However, given the expected alloy-dependent nature of $f$, Zhao et al. [21] have proposed a method to calculate $f$ as a function of the molar volumes of the alloy and oxide:

6 $$f_{z} = \frac{{2\sqrt {\frac{{V_{{{\text{ox}}}} }}{{V_{{\text{m}}} }}} }}{{\sqrt 6 + 2\sqrt {\frac{{V_{{{\text{ox}}}} }}{{V_{{\text{m}}} }}} }}.$$

Equation (6) was used to calculate a value of $f_{z}$ for subsequent determination of $N_{{{\text{Al}},z}}^{\left( 1 \right)}$ from Eq. (1). Note that $N_{{{\text{Al}}}}^{\left( 1 \right)}$ was calculated with the classical value of $f$ = 0.3 as reported by Rapp [15] and is distinguished by a different subscript. The stoichiometric ratio of oxygen to Al atoms in Al_2_O_3_, $\nu$, was taken to be 1.5.

Equation (2) was used to calculate the minimum critical concentration of solute Al required to maintain a continuous Al_2_O_3_ scale at 800 °C and 1000 °C in air for the experimental Alloy X and commercial alloys where values of $k_{{\text{p}}}$ were available in the literature, respectively. For Alloy X, the $k_{{\text{p}}}$ value was acquired by fitting Eq. (7) to the experimentally measured TGA data with the MATLAB Curve Fitting Tool:

7 $$\left( {\Delta m} \right)^{2} = k_{{\text{p}}} t$$

where $\Delta m$ is the specific mass change (mg/cm^2^), $k_{{\text{p}}}$ is the parabolic oxidation rate constant (mg^2^/cm^4^ h), and $t$ is the time (h). It is noted that the actual exponent value could deviate from 2 if oxidation does not follow parabolic kinetics. However, the original derivation of Eq. (2) [17] assumed a parabolic rate law and therefore requires the mathematical fitting of the $k_{{\text{p}}}$ value to be performed with an exponent value of 2. The total diffusivity of Al ($D_{{{\text{Al}}}}^{{\text{T}}}$) as calculated in *Thermo-Calc* was used for all the investigated alloys. For the remaining parameters ($V_{{\text{m}}}$, $\nu$), identical values incorporated in Eq. (1) were used.

## Results

### Wagner Model Results

The solubility and diffusivity of oxygen in solid Ni and the diffusivity of Al in solid Ni were calculated at 800 °C and 1000 °C with Eqs. (3–5) (Table 2).

**Table 2:** Solubility and diffusivity of oxygen in solid nickel at 800 °C/1000 °C and diffusivity of Al in solid nickel at 800 °C/1000 °C

Parameter	800 °C	1000 °C
$N_{{\text{O}}}^{{\text{S}}}$ (at. fr.)	1.75 × 10^−4^	4.60 × 10^−4^
$D_{{\text{O}}}$ (cm^2^/s)	7.07 × 10^−10^	3.45 × 10^−9^
$D_{{{\text{Al}}}}$ (cm^2^/s)	8.34 × 10^−13^	6.69 × 10^−11^

Using the parameters calculated in Table 2, the theoretical minimum critical concentration of Al (at%) required for external oxidation, $N_{{{\text{Al}}}}^{\left( 1 \right)}$, at 800 °C/1000 °C was calculated from Eq. (1) and is presented in Table 3 for the investigated alloys. In addition, the approach proposed by Zhao et al. [21] in Eq. (6) was used to calculate the volume fraction of internal oxide, $f_{z}$, which was subsequently used to calculate $N_{{{\text{Al}},z}}^{\left( 1 \right)}$.

**Table 3:** Calculated results of the minimum critical concentration of Al required for external oxidation at 800 °C/1000 °C for Alloy X and commercial alloys

Alloy	Oxidation type	$V_{{\text{m}}}$(cm^3^/mol)	$f_{z}$(at%)	$N_{{{\text{Al}},z}}^{\left( 1 \right)}$ (at%)	$N_{{{\text{Al}}}}^{\left( 1 \right)}$(at%)	$x_{{{\text{Al}}}}$ (at%)	$x_{{{\text{Al}}}}$ > $N_{{{\text{Al}},z}}^{\left( 1 \right)}$	$x_{{{\text{Al}}}}$ > $N_{{{\text{Al}}}}^{\left( 1 \right)}$
Alloy X	–	7.20	60.61	16.26	11.44	11.30	No	No
Rene N5	Al_2_O_3_ formers (800 °C)	7.16	60.68	16.23	11.41	13.90	No	Yes
PWA 1484		7.17	60.66	16.24	11.42	12.91	No	Yes
CMSX-4		7.16	60.68	16.23	11.41	12.59	No	Yes
Waspaloy	Cr_2_O_3_ formers (800 °C)	7.21	60.60	16.27	11.45	2.94	No	No
IN 738		7.19	60.62	16.26	11.44	7.00	No	No
RR1000		7.24	60.55	16.30	11.47	6.35	No	No
Type I	Giggins and Pettit (1000 °C)	6.98	60.99	6.43	4.51	2.14	No	No
Type II		7.20	60.61	6.51	4.58	4.10	No	No
Type IIIA		7.26	60.51	6.53	4.60	11.77	Yes	Yes
Type IIIB		7.11	60.76	6.48	4.55	17.50	Yes	Yes

To compare with the results presented in Table 3 (which used the diffusivity of Al in pure Ni), the values of $N_{{{\text{Al}}}}^{\left( 1 \right)}$ and $N_{{{\text{Al}},z}}^{\left( 1 \right)}$ were also calculated by using the total diffusivity of Al $D_{{{\text{Al}}}}^{{\text{T}}}$ computed in *Thermo-Calc*. The results are presented in Table 4 for the investigated alloys.

**Table 4:** Determination of the minimum critical concentration of Al required for external oxidation at 800 °C/1000 °C and the total diffusivity of Al ($D_{{{\text{Al}}}}^{{\text{T}}}$) computed in Thermo-Calc

Alloy	Oxidation type	$D_{{{\text{Al}}}}^{{\text{T}}}$(cm^2^/s)	$N_{{{\text{Al}},z}}^{\left( 1 \right)}$(at%)	$N_{{{\text{Al}}}}^{\left( 1 \right)}$(at%)	$x_{{{\text{Al}}}}$(at%)	$x_{{{\text{Al}}}}$ > $N_{{{\text{Al}},z}}^{\left( 1 \right)}$	$x_{{{\text{Al}}}}$ > $N_{{{\text{Al}}}}^{\left( 1 \right)}$
Alloy X	–	1.88 × 10^−12^	10.82	7.61	11.30	Yes	Yes
Rene N5	Al_2_O_3_ formers (800 °C)	1.29 × 10^−12^	13.06	9.18	13.90	Yes	Yes
PWA 1484		1.20 × 10^−12^	13.55	9.53	12.91	No	Yes
CMSX-4		1.13 × 10^−12^	13.93	9.80	12.59	No	Yes
Waspaloy	Cr_2_O_3_ formers (800 °C)	3.18 × 10^−13^	26.36	18.54	2.94	No	No
IN 738		6.32 × 10^−13^	18.67	13.14	7.00	No	No
RR1000		6.69 × 10^−13^	18.20	12.81	6.35	No	No
Type I	Giggins and Pettit (1000 °C)	2.25 × 10^−11^	11.08	7.77	2.14	No	No
Type II		4.33 × 10^−11^	8.09	5.69	4.10	No	No
Type IIIA		2.33 × 10^−10^	3.50	2.47	11.77	Yes	Yes
Type IIIB		1.24 × 10^−10^	4.75	3.34	17.50	Yes	Yes

The $N_{{{\text{Al}},z}}^{\left( 1 \right)}$ results are also included for comparison

Using the same parameters calculated in Table 2, the theoretical critical concentrations of Al (at%), $N_{{{\text{Al}}}}^{\left( 1 \right)}$ and $N_{{{\text{Al}},z}}^{\left( 1 \right)}$, required for external oxidation from 750 to 1050 °C with Eq. (1) for Alloy X are presented in Table 5.

**Table 5:** Calculated results of the minimum critical concentrations of Al, $N_{{{\text{Al}}}}^{\left( 1 \right)}$ and $N_{{{\text{Al}},z}}^{\left( 1 \right)}$, required for external oxidation from 750 to 1050 °C for Alloy X, along with the parameters required to determine these values

Alloy X	750 °C	800 °C	850 °C	900 °C	950 °C	1000 °C	1050 °C
$V_{{\text{m}}}$ (cm^3^/mol)	7.18	7.20	7.21	7.23	7.25	7.27	7.29
$N_{{\text{O}}}^{{\text{S}}}$ (at.fr. × 10^−4^)	1.29	1.75	2.30	2.95	3.72	4.60	5.59
$D_{{\text{O}}}$ (cm^2^/s)	4.32 × 10^−10^	7.07 × 10^−10^	1.11 × 10^−9^	1.67 × 10^−9^	2.44 × 10^−9^	3.45 × 10^−9^	4.76 × 10^−9^
$D_{{{\text{Al}}}}$ (cm^2^/s)	2.13 × 10^−13^	8.34 × 10^−13^	2.89 × 10^−12^	9.00 × 10^−12^	2.56 × 10^−11^	6.68 × 10^−11^	1.63 × 10^−10^
$f_{z}$ (at%)	60.64	60.61	60.59	60.56	60.53	60.50	60.47
$N_{{{\text{Al}},z}}^{\left( 1 \right)}$ (at%)	21.61	16.26	12.56	9.91	7.98	6.54	5.43
$N_{{{\text{Al}}}}^{\left( 1 \right)}$ (at%)	15.20	11.44	8.84	6.98	5.62	4.60	3.83
$x_{{{\text{Al}}}}$ (at%)	11.30	11.30	11.30	11.30	11.30	11.30	11.30
$x_{{{\text{Al}}}}$ > $N_{{{\text{Al}},z}}^{\left( 1 \right)}$	No	No	No	Yes	Yes	Yes	Yes
$x_{{{\text{Al}}}}$ > $N_{{{\text{Al}}}}^{\left( 1 \right)}$	No	No	Yes	Yes	Yes	Yes	Yes

Given that the transition from internal to external Al_2_O_3_ formation is expected at higher temperatures, it is therefore important to assess the ability of the investigated alloys to maintain an external scale of Al_2_O_3_. To this end, the theoretical minimum critical concentrations of Al, $N_{{{\text{Al}}}}^{\left( 2 \right)}$, required for maintaining the external Al_2_O_3_ scale at 800 °C and 1000 °C were calculated from Eq. (2) and are presented in Table 6 for the investigated alloys.

**Table 6:** Calculated results of the minimum critical concentration of Al, $N_{{{\text{Al}}}}^{\left( 2 \right)}$, required for maintaining external oxide growth at 800 °C/1000 °C for Alloy X and commercial alloys

Alloy	Oxidation type	$V_{{\text{m}}}$(cm^3^/mol)	$k_{{\text{p}}}$(mg^2^/cm^4^ h)	$N_{{{\text{Al}}}}^{\left( 2 \right)}$(at%)	$x_{{{\text{Al}}}}$(at%)	$x_{{{\text{Al}}}}$ > $N_{{{\text{Al}}}}^{\left( 2 \right)}$
Alloy X	–	7.20	7.48 × 10^−5^	2.79	11.30	Yes
PWA 1484	Al_2_O_3_ formers (800 °C)	7.17	2.52 × 10^−4^ [33]	6.40	12.91	Yes
CMSX-4		7.16	2.20 × 10^−5^ [34]	1.94	12.59	Yes
Waspaloy	Cr_2_O_3_ formers (800 °C)	7.21	3.60 × 10^−4^ [35]	14.93	2.94	No
IN 738		7.19	3.60 × 10^−4^ [36]	10.56	7.00	No
RR1000		7.24	9.12 × 10^−4^ [29]	16.44	6.35	No
Type I	Giggins and Pettit (1000 °C)	6.98	1.44 [1]	108.57	2.14	No
Type II		7.20	2.88 × 10^−2^ [1]	11.43	4.10	No
Type IIIA		7.26	9.00 × 10^−4^ [1]	0.88	11.77	Yes
Type IIIB		7.11	9.00 × 10^−4^ [1]	1.18	17.50	Yes

Oxidation rate constants were obtained or calculated from literature sources where listed

### Cross-Sectional Oxide Analysis

**Fig. 1:**
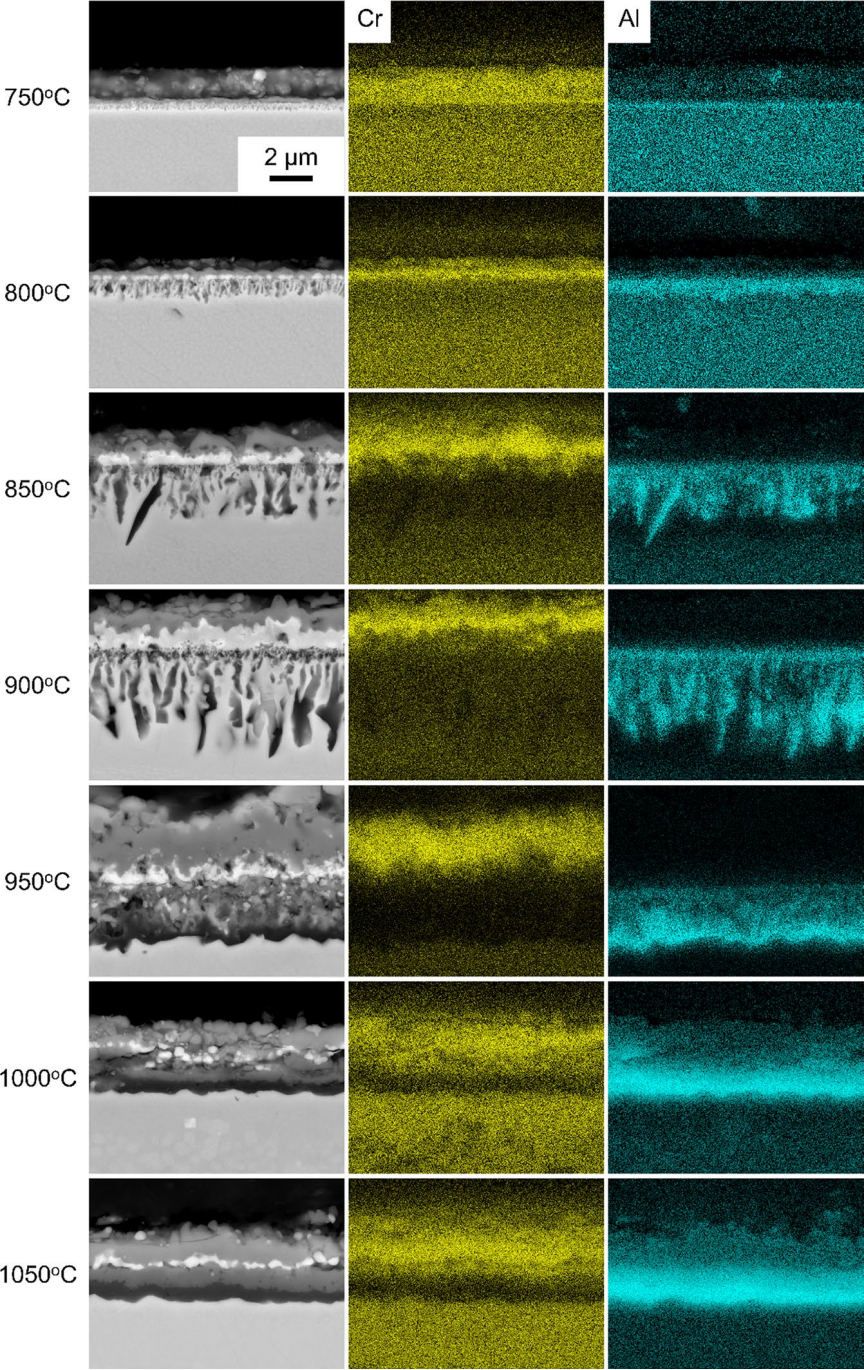
Representative BSE SEM images of Alloy X cross sections after exposure to air from 750 to 1050 °C (left). Corresponding EDX elemental concentration maps for Cr and Al are provided in addition to the BSE SEM images

The BSE micrographs of Alloy X cross sections after exposures at 750–1050 °C in air for 100 h are presented in Fig. 1 and accompanied by corresponding EDX elemental concentration maps of Cr and Al. A dense and continuous Cr_2_O_3_ scale was observed at all the investigated temperatures. From 750 to 900 °C, discontinuous Al_2_O_3_ intrusions underneath the Cr_2_O_3_ scale could be identified. The depth of the Al_2_O_3_ intrusions increased proportionately with temperature and reached a total value of ~ 4 µm. At 950 °C, a transition was observed where a largely continuous scale of Al_2_O_3_ formed underneath the external Cr_2_O_3_ scale. Sporadic regions of discontinuous Al_2_O_3_ intrusions were also observed, but these were not considered to be representative of the majority of the cross section. From 1000 to 1050 °C, a continuous Al_2_O_3_ scale was also observed and did not show any appreciable thickening compared with the sample exposed at 950 °C.

**Fig. 2:**
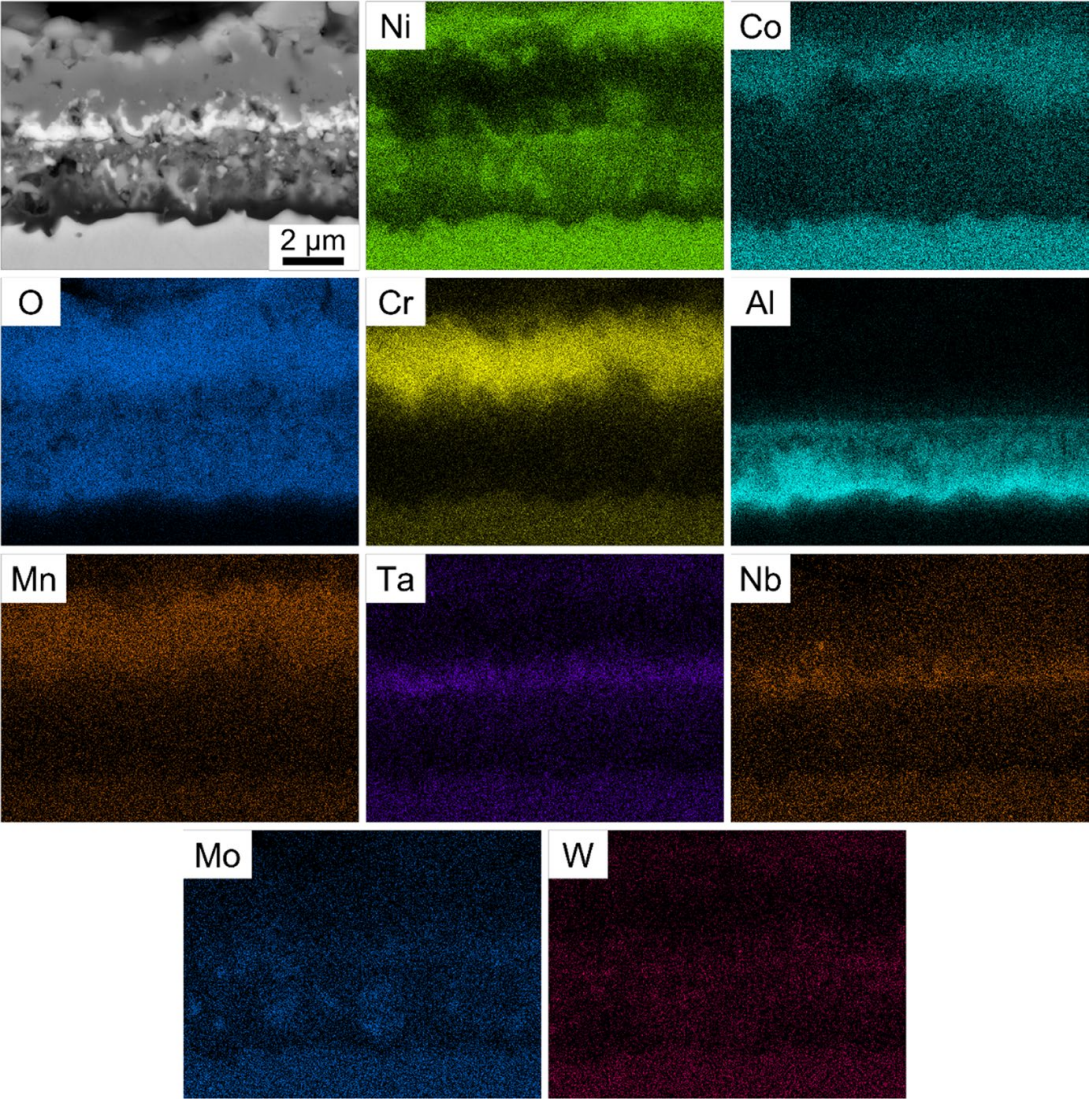
Cross-sectional BSE SEM image (top left) and corresponding EDX elemental concentration maps of Alloy X following oxidation in air at 950 °C for 100 h

As exposure at 950 °C for 100 h appeared to correspond with a transition in oxidation behaviour, this sample was examined in greater detail. The BSE cross-sectional images and associated EDX elemental concentration maps obtained from Alloy X after exposure at 950 °C for 100 h are presented in Fig. 2. As shown in Fig. 1, a solid external scale of Cr_2_O_3_ was observed (mid-grey in the BSE image). However, significant concentrations of Mn and Co were also detected in the external oxide layer, overlapping with regions of high Cr concentration. Light contrast intrusions enriched in Ta and Nb were observed beneath the Cr_2_O_3_ scale, which corresponded with the light contrast layer observed in the BSE image. The subscale consisted of primarily Ni with intermittent inclusions of Mo and W.

### Thermogravimetric Analysis Measurements

**Fig. 3:**
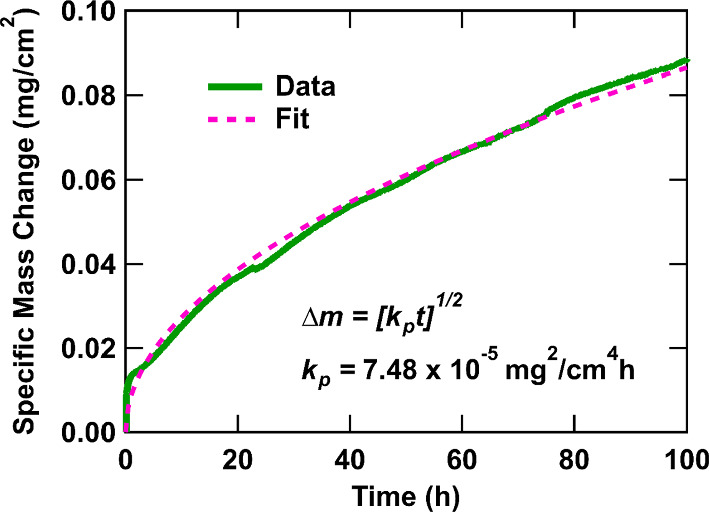
TGA specific mass change for Alloy X during oxidation at 800 °C for 100 h in air (green). The corresponding fit with Eq. (7) is concurrently displayed (magenta)

The specific mass change recorded using TGA for Alloy X during isothermal oxidation at 800 °C for 100 h is presented in Fig. 3. Within the first hour, Alloy X experienced a rapid initial increase in mass where it reached approximately 0.013 mg/cm^2^. After this point, the specific mass change slowed and reached approximately half (0.045 mg/cm^2^) of its overall specific mass change value after 30 h. Alloy X reached a final observed specific mass change (0.088 mg/cm^2^) after 100 h. The general curve profile is consistent with parabolic oxidation kinetics and a $k_{{\text{p}}}$ value of 7.48 × 10^−5^ mg^2^/cm^4^ h, 95% CI [7.46, 7.48 × 10^−5^] was calculated with Eq. (7). The 95% confidence bounds were computed with the MATLAB Curve Fitting Tool using Student’s cumulative *t*-distribution function. Minor oscillations were also observed in the TGA data that follow a roughly 24-h cycle, which was associated with day/night temperature variations within the laboratory.

## Discussion

### Input Parameters

The calculated $N_{{\text{O}}}^{{\text{S}}}$ value is similar to other reported oxygen solubilities (e.g. 10^−4^ [15], 1.9 × 10^−4^ [37]). The results also agree with the $N_{{\text{O}}}^{{\text{S}}}$ values reported in [38] which range from 6.9 × 10^−4^ (800 °C) to 4.8 × 10^−4^ (1100 °C) in Ni–Al alloys. Nesbitt [16] calculated a value of 2.4 × 10^−5^ by employing a combination of Sievert’s and Henry’s laws [16]. In the same work, Nesbitt also observed a $N_{{\text{O}}}^{{\text{S}}}$ value of 4.4 × 10^−4^ for the case where an external scale initially exists, which was taken from [37] for a NiO dissociation pressure at the oxide–metal interface (1 × 10^−7^ atm). It is noted that Nesbitt investigated the isothermal oxidation of Ni–Cr–Al alloys at 1200 °C, which could explain the disparity in the magnitude of the calculated $N_{{\text{O}}}^{{\text{S}}}$ value in this study. Given that multiple literature sources consistently report oxygen solubility in Ni alloys to be on the order of 10^−4^, reasonable confidence is placed in the calculated value of $N_{{\text{O}}}^{{\text{S}}}$ in this work.

The calculated $D_{{\text{O}}}$ value (7.07 × 10^−10^ cm^2^/s) agrees reasonably and falls within the same order of magnitude as other reported values [39, 40] when considering the differences in investigated temperatures. However, it differs from [16] and [38]. In the former, a much higher temperature was investigated (1200 °C), which would give a significantly higher diffusion constant. In the latter, a value of 6.3 × 10^−12^ cm^2^/s was quoted for the diffusion of O in Ni at 800 °C from [40]. However, it is unclear where this value was reported in the original paper. In general, the $D_{{\text{O}}}$ values vary significantly in the literature due to different calculation methods and assumptions. Despite the wide variation, the calculated $D_{{\text{O}}}$ value in this work appears to align with reported values within sensible margins (i.e. order of 10^−10^). Barlow and Grundy [41] reported a $D_{{\text{O}}}$ value of 8.38 × 10^−11^ cm^2^/s for the diffusion of O in Ni at 800 °C by studying internal oxidation, which is an order of magnitude smaller than this work. However, this discrepancy could be explained by noting that they based their calculation of $D_{{\text{O}}}$ on the solubility values of O reported in [37], whereas [31] calculated the solubility of O independently using electrochemical methods. However, the pre-exponential terms in [31] and [41] differed significantly despite a close agreement in the magnitudes of the diffusivities [31]. Considerable variation is observed within the literature concerning the determination of Arrhenius-style equation parameters for the calculation of O diffusivity [42–45] and mainly depends on whether O diffuses through the alloy in a substitutional or interstitial manner. The relatively large activation energies reported for O diffusion suggest that the oxidation of alloys is only partially dependent on the diffusion of O [31]. However, a more recent computational study [46] calculated similar Arrhenius parameters as [31] and argued that diffusivities measured by electrochemical methods were more reliable than alternative studies that were dependent on the O solubility. The solubility limits of O in various oxides encountered in complex alloys are proposed as a necessary consideration for a representative characterisation of $D_{{\text{O}}}$.

In [31], Eqs. (3) and (4) were derived to calculate $N_{{\text{O}}}^{{\text{S}}}$ and $D_{{\text{O}}}$, respectively, by assuming a system where Ni and O exist in equilibrium with NiO. Correspondingly, the partial pressure of oxygen (or dissociation pressure) is expected to be significantly higher than a scenario where an existing external Cr_2_O_3_ scale is established. Using the dissociation pressure of Cr_2_O_3_ would result in a smaller oxygen partial pressure and $N_{{\text{O}}}^{{\text{S}}}$ value, thereby reducing the $N_{{{\text{Al}}}}^{\left( 1 \right)}$ criterion. However, it can be argued that Eqs. (3) and (4) would be appropriate in the early stage of oxidation (i.e. transient) where NiO can dominate scale formation. It is only when a continuous Cr_2_O_3_ scale begins to form that the expressions become less compatible. While calculating the dissociation pressure of Cr_2_O_3_ from standard Gibbs free energies is relatively straightforward, it would be more challenging to implement alongside the work of Park and Altstetter [31]. A potential approach would be to experimentally identify when a Cr_2_O_3_ scale begins to form for a given alloy and then calculate a time-weighted average value of $N_{{\text{O}}}^{{\text{S}}}$ and $D_{{\text{O}}}$ for use in Eq. (1). Further work is needed to investigate the effect of existing/compound oxide scales on the dissociation pressures and subsequent oxygen solubility.

The calculated $D_{{{\text{Al}}}}$ value is similar to other reported values [47, 48]. Notably, the $D_{{{\text{Al}}}}$ value is significantly higher than in [49]. However, Allison and Samelson [32] attributed this discrepancy to smaller grain sizes in their work (approximately 45 µm) when compared to the results of [49] (0.5–10 mm), which could lead to more grain boundaries and higher diffusion rates. As a result, the work in [32] is consistent with the expected range of grain sizes of 49 µm and 13–89 µm in coarse-grained RR1000 [43] and Waspaloy [50], respectively. The average grain size for as-cast IN 738 is reported to be over 100 µm [51], which at first would make Eq. (5) seem invalid in favour of [49]. However, the average grain sizes investigated in [49] were significantly larger than the reported grain sizes in IN 738. As a result, IN 738 can be argued to be more similar to the samples investigated in [32] and therefore a higher $D_{{{\text{Al}}}}$ is expected. Moreover, the method still correctly predicted the Al_2_O_3_-forming nature of IN 738, which suggests that the method proposed in [32] may still be valid for alloys with relatively large grains (> 100 µm). Further investigation into the effect of grain boundary diffusion in the calculation of $D_{{{\text{Al}}}}$ in commercial alloys is needed to refine the prediction of protective oxide scales.

Table 4 suggests that the calculated $D_{{{\text{Al}}}}^{{\text{T}}}$ values give reasonable predictions of Al_2_O_3_-forming behaviour. However, except for Waspaloy and RR1000, the $D_{{{\text{Al}}}}^{{\text{T}}}$ values are generally greater than the value calculated with Eq. (5) from [32]. This is generally expected since the contributions of the other components in the alloy to the diffusion of Al are captured when using *Thermo-Calc*, whereas Eq. (5) assumes a Ni–Al binary system. The $D_{{{\text{Al}}}}^{{\text{T}}}$ values for the Cr_2_O_3_ formers and the GP Type I and Type II alloys are within the same order of magnitude as $D_{{{\text{Al}}}}$ calculated with Eq. (5), showing good agreement. It is unclear why the $D_{{{\text{Al}}}}^{{\text{T}}}$ values of Waspaloy and RR1000 were smaller than the value predicted with Eq. (5) despite accounting for the additional diffusivity contributions from other components in the alloys; as such, more in-depth investigation is needed. In general, the $N_{{{\text{Al}},z}}^{\left( 1 \right)}$ value obtained using $D_{{{\text{Al}}}}^{{\text{T}}}$ agrees with the results in Table 3. However, the reverse is observed for Rene N5 where the $f_{z}$ value calculated by Eq. (6) proposed in [21] resulted in a $N_{{{\text{Al}},z}}^{\left( 1 \right)}$ value of 0.1306, which was marginally lower than the $x_{{{\text{Al}}}}$ value of Rene N5.

The calculated $f_{z}$ values ranged from 0.6051 to 0.6099 and were substantially higher than $f$ = 0.3 as reported in [15]. This overestimation is expected based on [21] where a consistent overestimation was observed in Ag–In and Fe–Si alloys. Equation (6) considers the “blocking effect” where, as time progresses, the oxides formed within the internally oxidised zone act as increasingly stronger barriers, impeding the diffusion of O into the alloy. However, Zhao et al. [21] noted that their proposed model did not account for the morphology of the oxides nor the degree of supersaturation. When used to subsequently calculate the $N_{{{\text{Al}},z}}^{\left( 1 \right)}$, the model did not predict the oxidation behaviour of the known Al_2_O_3_ formers Rene N5, PWA 1484, and CMSX-4. However, the use of $f_{z}$ was successful in predicting the GP Type IIIA and Type IIIB Ni–Cr–Al alloys. One possible reason is that the findings in [1] showed the gradual coarsening of Al_2_O_3_ particles beneath the Cr_2_O_3_ scale, indicating a transition from a Type II to a Type III Al_2_O_3_-forming alloy. This observation aligns with the assumptions in [21] regarding the “blocking effect”. However, it is not clear why $f_{z}$ failed to predict the known commercial Al_2_O_3_-forming alloys. This may be attributed to the commercial alloys having significantly more complex chemistries than the Ni–Cr–Al alloys investigated in [1]. In addition, the different morphologies and competing effects during early stages oxidation were not accounted for in the model proposed in [21].

### Commercial Alloys

**Fig. 4:**
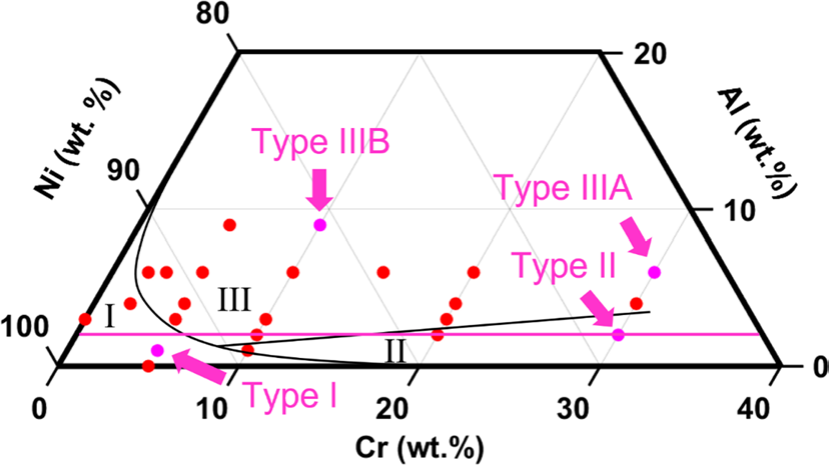
Ternary composition diagram map of Ni–Cr–Al alloys at 1000 °C outlining the regions for Type I, Type II, and Type III oxidation behaviour. The magenta arrows and markers identify the compositions that were investigated in this study, and the magenta line approximately indicates the calculated $N_{{{\text{Al}}}}^{\left( 1 \right)}$ value (4.5 at% or 2.2 wt%) in this study. Based on the original diagram from Giggins and Petit [1]

Table 3 shows that the modelling approach appears to correctly predict the Al_2_O_3_-forming behaviour of the Rene N5, PWA 1484, and CMSX-4 alloys by calculating $N_{{{\text{Al}}}}^{\left( 1 \right)}$ values that were lower than the respective $x_{{{\text{Al}}}}$ values. For the known Cr_2_O_3_-forming alloys Waspaloy, IN 738, and RR1000, the calculated $N_{{{\text{Al}}}}^{\left( 1 \right)}$ values were indeed larger than the respective $x_{{{\text{Al}}}}$ values. Furthermore, the model also correctly predicted that the GP Type I and Type II alloys [1] failed to produce an external Al_2_O_3_ scale. Equally, the model correctly predicted the GP Type IIIA and Type IIIB alloys as Al_2_O_3_-forming compositions. For the GP Type IIIA and Type IIIB alloys, the $N_{{{\text{Al}}}}^{\left( 1 \right)}$ values are relatively low compared to $x_{{{\text{Al}}}}$. The results are in excellent agreement with the empirically developed ternary diagram at 1000 °C originally reported in [1] and shown in Fig. 4.

In Fig. 4, the $N_{{{\text{Al}}}}^{\left( 1 \right)}$ values calculated for GP Type IIIA and Type IIIB oxidation behaviour are relatively far from the boundary line separating Type II and Type III oxidation behaviour, suggesting that these compositions have higher concentrations of Al than are required to form an external scale of Al_2_O_3_. Moreover, the boundary line at the interface of Type II and Type III regions appears to be reasonably consistent with the calculated $N_{{{\text{Al}}}}^{\left( 1 \right)}$ values for these alloys (approximately 4.5 at% Al or 2.2 wt%), demarcated by the magenta line.

Commercial alloys examined in this study generally satisfied Wagner's criterion [Eq. (2)] for sustaining continuous growth of an Al_2_O_3_ scale. The Al_2_O_3_-forming alloys, PWA 1484 and CMSX-4, had lower $N_{{{\text{Al}}}}^{\left( 2 \right)}$ values of 6.40 and 1.93 at%, respectively (Table 6), compared with their $N_{{{\text{Al}}}}^{\left( 1 \right)}$ values of 9.53 and 16.24 at%, respectively (Tables 3 and 4). These results suggest that Al concentration and diffusion to the metal–oxide interface are sufficient for maintaining continuous Al_2_O_3_ scale growth. In contrast, the Cr_2_O_3_-forming alloys exhibited higher $N_{{{\text{Al}}}}^{\left( 2 \right)}$ values (10.56–16.60 at%), suggesting insufficient Al concentrations to sustain continuous Al_2_O_3_ scale growth. This could be due to lower Al diffusivities ($D_{{{\text{Al}}}}$) in the Cr_2_O_3_-forming alloys (× 10^−13^) compared with the Al_2_O_3_-forming alloys (× 10^−12^). Waspaloy and RR1000 alloys had significantly higher $N_{{{\text{Al}}}}^{\left( 2 \right)}$ values (14.93 and 16.60 at%, respectively) than their $N_{{{\text{Al}}}}^{\left( 1 \right)}$ values (11.45 and 26.36 at%, respectively), supporting their inability to form continuous Al_2_O_3_ scales. Therefore, even if an Al_2_O_3_ scale is established on these alloys under different conditions, it would not be possible to regenerate the Al_2_O_3_ scale in the case of spallation. However, IN 738 exhibited a lower $N_{{{\text{Al}}}}^{\left( 2 \right)}$ value (10.56 at%) compared to its $N_{{{\text{Al}}}}^{\left( 1 \right)}$ values (11.44–16.26 at%), which could be attributed to its higher Al concentration (7.00 at%) and lower Mo concentration (1.03 at%). The unrealistic $N_{{{\text{Al}}}}^{\left( 2 \right)}$ value (108.57 at%) calculated for the GP Type I alloy (Ni–5Cr–1Al) resulted from an extraordinarily high $k_{{\text{p}}}$ value (1.44 mg^2^/cm^4^ h) which was reported in [1]. This $k_{{\text{p}}}$ value has a near-exact agreement with the $k_{{\text{p}}}$ of pure NiO scale formation [52], which is expected since a Type I alloy is generally associated with non-protective NiO scale formation. Since NiO formation is typically associated with linear kinetics, Eq. (2) shows that a large $k_{{\text{p}}}$ value can outweigh Al diffusional effects and is therefore unsuitable for Type I alloys.

The calculated $N_{{{\text{Al}}}}^{\left( 2 \right)}$ values (0.88–11.43 at%) for the GP Type II (Ni–30Cr–2Al), Type IIIA (Ni–30Cr–6Al), and Type IIIB (Ni–10Cr–9Al) alloys were reasonable. The Type II alloy not only has slightly more Al than the Type I alloy but significantly more Cr, highlighting the significant protection afforded by Cr_2_O_3_ and possible synergistic effects. However, Eq. (2) predicted relatively low $N_{{{\text{Al}}}}^{\left( 2 \right)}$ values (0.88–1.18 at%) compared to $N_{{{\text{Al}}}}^{\left( 1 \right)}$ (2.47–6.53 at%) for the Type IIIA and Type IIIB alloys. It is unclear why $N_{{{\text{Al}}}}^{\left( 2 \right)}$ was extremely low for these alloys, but one explanation is that the diffusivity $D_{{{\text{Al}}}}$ at 1000 °C is much higher than at 800 °C, which could compensate for the slightly increased $k_{{\text{p}}}$ rates [1].

### Alloy X

The results shown in Fig. 1 indicate a transition from Type II to Type III oxidation in the 900–950 °C range for Alloy X. A Cr_2_O_3_ scale formed at all temperatures with relatively consistent thickness. Initially, Al_2_O_3_ formed discontinuously at 750 °C and gradually increased to an approximate thickness of 4–5 μm at 900 °C. However, at 950 °C, a dramatic change occurred with a continuous Al_2_O_3_ scale forming beneath an external Cr_2_O_3_ scale and intermediate subscale of complex oxides. This suggests that the kinetics at 950 °C are sufficiently high such that Al can readily diffuse to the metal–subscale interface, aligning with the mechanisms outlined in [2]. Importantly, the Al_2_O_3_ scale remains continuous at 1000 °C and 1050 °C, providing further evidence that the transition temperature from Type II to Type III oxidation lies in the 900–950 °C range for Alloy X.

Table 5 shows that the Wagner criterion for external oxidation, using $f$ values by [15] and $f_{z}$ values by [21], predicts the transition temperature of Alloy X to be 850 °C and 900 °C, respectively. This is consistent with the experimental results at 800 °C (Fig. 1) where Alloy X does not exhibit Type III oxidation. However, the experimentally determined transition temperature (950 °C) for Alloy X exceeds the Wagner criterion predictions using both internal volume fraction calculation methods, suggesting an underestimation of the Type III oxidation transition by approximately 50–100 °C. At 900 °C, Alloy X exhibits primarily Type II oxidation but shows isolated regions of Type III oxidation with semi-continuous Al_2_O_3_ scales. The higher $f_{z}$ values, almost twice as high as the $f$ values, are putatively consistent with the observed complex subscale of oxides and suggest that the approach proposed in [21] shows some validity for Alloy X.

The increased oxide volume fraction of the complex oxide subscale at 900–950 °C (Fig. 2), combined with the external Cr_2_O_3_ scale, may have inhibited oxygen ingress and facilitated Al diffusion to the subscale to establish a continuous Al_2_O_3_ scale. The enrichment of Ta- and Nb-based oxides beneath the external Cr_2_O_3_ scale confirms similar findings in related alloys [53, 54] and could be attributed to Cr-induced uphill diffusion in the Cr-depleted subscale. The Mo and W inclusions in the subscale at 950 °C have also been reported in similar alloys [55], suggesting significant transient oxidation may have occurred before continuous Al_2_O_3_ scale formation when compared to the lower investigated temperatures. This is supported by Fig. 1 where the absence of a complex subscale at 850–900 °C may have resulted in discontinuous Al_2_O_3_ intrusions despite an actual Al concentration (11.30 at%) that surpasses the minimum values (8.84 at% and 6.98 at%) predicted by the Wagner criterion. The limitation of the Wagner criterion to accurately determine the Type III oxidation transition temperature could be attributed to its failure to account for compound oxide scale effects.

The sensitivity of the Wagner criterion to its input parameters was examined by using different values [16, 31, 37, 38, 41, 47, 48, 56] for $N_{{\text{O}}}^{{\text{S}}}$, $D_{{\text{O}}}$, and $D_{{{\text{Al}}}}$ to calculate $N_{{{\text{Al}}}}^{\left( 1 \right)}$ for Alloy X at 800 °C. Values of $N_{{\text{O}}}^{{\text{S}}}$ < 2 × 10^−4^, $D_{{\text{O}}}$ < 7 × 10^−10^ cm^2^/s, and $D_{{{\text{Al}}}}$ > 8 × 10^−13^ cm^2^/s gave a value of $N_{{{\text{Al}}}}^{\left( 1 \right)}$ < 11.30 at%. The wide variation in the reported values and calculation methods for these parameters occasionally led to incorrect predictions of Type III behaviour in Alloy X. In addition, the calculated value of $D_{{{\text{Al}}}}^{{\text{T}}}$ with *Thermo-Calc* (1.88 × 10^−12^ cm^2^/s) in Table 4 was an order of magnitude higher than the other values used in this study and resulted in an erroneous prediction at 800 °C. Overall, the Wagner model shows high sensitivity to diffusivity and oxygen solubility parameters. Further research is needed to identify optimal methods for parameter calculation and assess the applicability of assumptions based on simple binary systems to superalloys with complex compositions.

The Al concentration of Alloy X exceeds the calculated $N_{{{\text{Al}}}}^{\left( 2 \right)}$ value, suggesting that it could maintain an established Al_2_O_3_ scale. Notably, its $D_{{{\text{Al}}}}^{{\text{T}}}$ value is on the same order of magnitude (× 10^−12^) as the commercial Al_2_O_3_ formers and an order of magnitude higher than the commercial Cr_2_O_3_ formers (× 10^−13^). Its $k_{{\text{p}}}$ value (7.48 × 10^−5^ mg^2^/cm^4^ h) is also an order of magnitude lower than the commercial Cr_2_O_3_ formers despite relying on the formation of Cr_2_O_3_ for oxidation resistance. This is supported by the TGA results for Alloy X in Fig. 3, where its final specific mass change is substantially lower than that reported for RR1000 at 800 °C [29]. The higher diffusivity of Al in Alloy X compared with the commercial Cr_2_O_3_ formers can be attributed to its higher Al concentration (11.30 at%), which could facilitate rapid diffusion to the metal–oxide interface. In addition, Alloy X is the only investigated alloy that contains an appreciable concentration of Mn, which has been previously reported to improve oxidation resistance [57, 58]. The parabolic growth rate of an oxide scale is typically associated with the solid-state diffusion of species through a single scale [59]. However, Fig. 1 shows that Alloy X formed multiple oxides. Therefore, the $k_{{\text{p}}}$ rate constant in this study should be considered as an apparent value rather than an actual value. Instantaneous rate constants may be more suitable for describing the growth of individual oxide scales.

The mixed results for $N_{{{\text{Al}}}}^{\left( 1 \right)}$ (Tables 3 and 4) suggest that the Al concentration of Alloy X may not be sufficient to instigate a transition from internal to external Al_2_O_3_ formation at 800 °C. However, Alloy X has an adequate Al concentration to satisfy the calculated $N_{{{\text{Al}}}}^{\left( 2 \right)}$ value (2.79 at%), suggesting its potential to maintain and regenerate a continuous Al_2_O_3_ scale if established under different conditions. For instance, a suitable pre-oxidation treatment at higher temperatures could lower the $N_{{{\text{Al}}}}^{\left( 1 \right)}$ value, enabling Alloy X to meet the transition criterion and sustain continuous Al_2_O_3_ formation at lower service temperatures.

It is acknowledged that oxide spallation in cyclic conditions is a concern for in-service operation. The uncertainty of Al_2_O_3_ scale regeneration and the potential formation of less-protective oxides could lead to break-away oxidation. In this regard, $N_{{{\text{Al}}}}^{\left( 2 \right)}$ considers the Al diffusion and oxidation rate constant as measures of the ability of an alloy to reform a protective Al_2_O_3_ scale after spallation. However, partial or complete spallation of the scale contradicts the assumption of an initially present continuous external scale for $N_{{{\text{Al}}}}^{\left( 2 \right)}$ to be applied. For partial spallation, it is approximated that regions of bare metal surrounded by continuous Al_2_O_3_ scales are formed on the alloy surface. For complete spallation, the entire alloy surface is exposed, necessitating re-compliance of the $N_{{{\text{Al}}}}^{\left( 1 \right)}$ criterion. However, it remains uncertain how scale reformation would proceed in these scenarios considering the formation of an Al-depleted region beneath the metal–oxide interface. Therefore, a comprehensive investigation of the cyclic oxidation of Alloy X is required to evaluate the resilience of Al_2_O_3_ scales formed during isothermal oxidation if it is to be considered for commercial applications.

The samples in this study were polished to a 1-µm diamond surface finish which is not representative of commercial turbine disc components. Since Wagner’s criteria assume ionic diffusion in the oxide scale is rate-controlling and reactions at the metal–surface and oxide–gas boundaries are in equilibrium, it is inferred that the surface morphology is ignored. Therefore, the polished samples allowed for a comparison with Wagner’s criteria and the literature as well as providing a uniform initial surface condition for reproducibility. However, it should be acknowledged that Alloy X, which showed regions of continuous Al_2_O_3_ scales, may possess a composition inclined to undergo Type III oxidation, wherein the influence of surface roughness on oxidation mechanisms could be significant. Further research is therefore warranted to explore the impact of surface roughness on the oxidation behaviour of Alloy X, to assess its commercial suitability.

## Conclusions

The Wagner criteria for the internal to external oxidation transition and the maintenance of an external Al_2_O_3_ scale were used to predict the critical concentration of Al required for continuous Al_2_O_3_ formation in experimental and commercial Ni-based superalloys from 750 to 1050 °C:

- The Wagner model successfully predicted the oxidation behaviour of known Al_2_O_3_-forming commercial superalloys and simpler Ni–Cr–Al systems outlined in [1].
- Testing the Wagner model on the experimental Alloy X at multiple temperatures gave an underprediction of the transition temperature by approximately 50–100 °C.
- The calculation of the internal oxide volume fraction ($f_{z}$) using the method proposed in [21] correctly predicted the oxidation behaviour of some Ni–Cr–Al alloys but not the commercial alloys, possibly due to complex oxide formation. It showed relatively better accuracy in predicting the transition temperature of Alloy X.
- The Wagner criteria exhibited high sensitivity to the solubility and diffusivity input parameters. The significant variability of these values in the literature was also discussed. Further research is needed to refine the accuracy of parameter calculation and selection, leading to improved prediction of oxidation behaviour in Ni-based superalloys.

## Supplementary Information

Below is the link to the electronic supplementary material.

Supplementary file1 (DOCX 20 KB)

## Data Availability

The underlying research data required to reproduce these findings are available from the University of Cambridge repository (https://doi.org/10.17863/CAM.95331) [60].

## Declarations

### Use of OpenAI API

ChatGPT (GPT-3, OpenAI’s large-scale language-generation model) was used to check grammar and improve conciseness in response to reviewer suggestions. JWXW reviewed, edited, and revised all text that was generated by ChatGPT to his own satisfaction, and the authors take ultimate responsibility for the content of this publication.
