# Supplementary material for: Evaluating Wagner Oxidation Criteria for Protective Al2O3 Scale Formation in Ni-Based Superalloys
Source: High Temp Corros Mater. 2023 Jun 26;100(1-2):85–108. doi: 10.1007/s11085-023-10163-5 (PMC10348012; doi:10.1007/s11085-023-10163-5)
Supplement: Supplementary file 1 — Supplementary file1 (DOCX 20 KB) [file 11085_2023_10163_MOESM1_ESM.docx]

Table 1 – Measured elemental composition of Alloy X with large-area energy dispersive X-ray (EDX) operated at 20 kV. The nominal composition is provided for comparison. Three representative sites throughout the cross-section of Alloy X were selected and subjected to EDX characterisation to calculate an average concentration value for each element. The light elements (carbon and boron) were excluded from the analysis but listed in the table for reference. For each site, a field-of-view with approximate dimensions of 52 x 40 μm^2^ and an acquisition time of 10 minutes were selected. Standard deviations for the actual measured values are provided.

| **Element (at.%)** | **Alloy X** | | | **Measured** | **Standard Deviation** | **Nominal** |
| --- | --- | --- | --- | --- | --- | --- |
|  | **Site 1** | **Site 2** | **Site 3** |  |  |  |
| **Ni** | 49.20 | 49.31 | 49.50 | **49.34** | 0.15 | **49.85** |
| **Co** | 19.62 | 19.78 | 19.55 | **19.65** | 0.12 | **19.36** |
| **Cr** | 13.06 | 13.15 | 13.07 | **13.09** | 0.05 | **12.74** |
| **Al** | 11.45 | 11.21 | 11.27 | **11.31** | 0.12 | **11.30** |
| **Mo** | 1.74 | 1.73 | 1.74 | **1.74** | 0.01 | **1.79** |
| **Ta** | 1.82 | 1.77 | 1.81 | **1.80** | 0.03 | **1.63** |
| **W** | 1.46 | 1.51 | 1.46 | **1.48** | 0.03 | **1.27** |
| **Nb** | 0.96 | 0.86 | 0.92 | **0.91** | 0.05 | **1.02** |
| **Mn** | 0.62 | 0.60 | 0.63 | **0.62** | 0.02 | **0.59** |
| **Zr** | 0.03 | 0.05 | 0.04 | **0.04** | 0.01 | **0.06** |
| **Ti** | 0.03 | 0.03 | 0.02 | **0.03** | 0.01 | **0.04** |
| **C** | - | - | - | **-** | - | **(0.15)** |
| **B** | - | - | - | **-** | - | **(0.2)** |
| **Total** |  |  |  | **100.00** |  |  |
